# Self-collected versus medic-collected sampling for human papillomavirus testing among women in Lagos, Nigeria: a comparative study

**DOI:** 10.1186/s12889-022-14222-5

**Published:** 2022-10-15

**Authors:** Ning Feng, Oliver Ezechi, Mabel Uwandu, Bowofoluwa Sharon Abimbola, Grace Deborah Vincent, Ifeoma Idigbe, Leona Chika Okoli, Mary Adesina, Jane Okwuzu, Rahaman Ademolu Ahmed, Judith Sokei, Joseph Ojonugwa Shaibu, Abidemi Esther Momoh, Omowunmi Sowunmi, Olaoniye Habeebat Labo-Popoola, POPGEC Team, Mfon Victoria Sunday, Janet Fayemi, Hannah Mfon Udoh, Mayokun Omidiji, Oluwatobi Ogundepo, Victor Ogbolu, Greg Ohihoin, Agatha David, Emily Nzeribe, Olufemi Olaleye, Xiao-ping Dong, Chika Kingsley Onwuamah

**Affiliations:** 1 Center for Global Public Health, Chinese Centre for Disease Control and Prevention, 155 Changbai Road, Changping District, Beijing, 102206 China; 2 Department of Clinical Sciences, Nigerian Institute of Medical Research, 6 Edmund Crescent, Yaba, Lagos, Nigeria; 3 Centre for Human Virology and Genomics, Department of Microbiology, Nigerian Institute of Medical Research, 6 Edmund Crescent, Yaba, Lagos, Nigeria; 4 Federal Medical Centre, 105 Orlu Road, Owerri, Imo State Nigeria; 5 Optimal Cancer Care Foundation Centre, Lagos, Nigeria; 6 National Institute for Viral Disease Control and Prevention, Chinese Center for Disease Control and Prevention, 155 Changbai Road, Changping District, Beijing, 102206 China

**Keywords:** HPV, Self-sampling, Medic-sampling, PCR, Sensitivity, Specificity, Accuracy

## Abstract

**Objective:**

To evaluate the feasibility and performance of self-collected vaginal swab samples for HPV screening among women in Lagos, Nigeria.

**Methods:**

A cross-sectional study was implemented from March to August 2020 among sexually active women. Study participants provided same-day paired vaginal swab samples. Medic-sampling and poster-directed self-sampling methods were used to collect the two samples per participant. A real-time PCR assay detected HPV 16, HPV 18, other-high-risk (OHR) HPV, and the human β-globin gene. The self-collected samples’ sensitivity, specificity, and accuracy were determined against the medic-collected samples using the MedCalc Online Diagnostic Calculator.

**Results:**

Of the 213 women aged 16 ~ 63-year-old recruited, 187 (88%) participants had concordant results, while 26 (12%) participants had discordant results. Among the 187 concordant results, 35 (19%) were HPV positive, 150 (80%) participants were HPV negative, and two (1%) were invalid. 18 (69%) out of the 26 discordant samples were invalid. The self-collected sample was invalid for 14 (54%) participants. Two (8%) medic-collected samples were invalid. Compared to the medic-collected sample, the self-collected sample was 89.80% (95% CI: 77.77 ~ 96.60%) sensitive and 98.21% (95% CI: 94.87 ~ 99.63%) specific, with an accuracy of 96.31% (95% CI: 92.87 ~ 98.40%). The mean age for HPV positive and negative participants were 39 and 40, respectively, with an ANOVA *p*-value of 0.3932. The stratification of HPV infection by the age group was not statistically significant (*P* > 0.05).

**Conclusions:**

With high accuracy of 96%, self-collected sampling is adequate when tested with real-time PCR and may increase the uptake of HPV testing. Though more self-collected samples were invalid than medic-collected samples, most likely due to poor collection, they could be identified for repeat testing. Future implementation can avoid this error with improved guidance and awareness.

**Supplementary Information:**

The online version contains supplementary material available at 10.1186/s12889-022-14222-5.

## Background

The Human Papillomavirus (HPV) is a common newly diagnosed sexually transmitted infection, prevalent among sexually active persons, and 80% of women will acquire this infection [1, 2]. HPV infection is responsible for more than 91% of cervical cancer [3]. The high-risk oncogenic HPV types (HPV 16 and HPV 18) are associated with more than 70% of cases of cervical cancer, and the low-risk HPV types (HPV 6 and 11) are associated with abnormal pap tests and genital warts [4, 5].

Cervical cancer is the fourth most common cancer in women globally, with 84 ~ 90% of the burden in low and middle-income countries (LMICs) [6]. The more significant disease burden is in sub-Saharan Africa (age-standardised incidence rate of 50/100,000 compared to 5/100,000 in high-income countries (HICs) [7]. Recent data reports that cervical cancer accounts for 7.5% of female cancer deaths, and 90% of these deaths occur in LMICs [6, 8]. Compared to more than 60% of women in HICs screened for cervical cancer, only about 20% of women in LMICs are screened for cervical cancer as standard cervical cancer screening tests are not readily available [9, 10]. A meta-analysis of HPV incidence among HIV-positive women in developing countries yielded a pooled prevalence of 63% from nineteen studies that recruited 8175 HIV-positive women, being 51.0% for high-risk HPV and 28% for low-risk HPV [11], With a prevalence of 18.6% and cervical cancer estimated at 5/1000 women, Nigeria’s burden of HPV is high [11, 12]. The World Health Organisation recommends women between 30 to 49 years should screen with more sensitive tests that detect HPV in cervical or vaginal samples as HPV has a long preclinical phase [13, 14]. Due to the limiting barriers in LMICs, visual inspection under acetic acid is common though it performs poorly compared to HPV deoxyribonucleic acid (DNA) testing [15–17].

In Nigeria, cancer screening is available in few public and private health facilities, even as HPV DNA testing is recommended globally as a better screening method for cervical cancer. However, the uptake of cervical cancer screening in most Nigerian populations is still low [18]. Low uptake is due to many reasons, especially as most people wait to experience significant disease symptoms before visiting healthcare centres. Other obstacles causing the low uptake of screening services include unwillingness to be examined by male healthcare workers, fear of stigmatisation if positive, fear of hospital-acquired infection, and need for husbands’ approval [19, 20]. Strategies employed to increase the uptake of cervical screening in Nigeria include creating awareness, free or subsidised HPV testing programs, and involvement of the male gender in the sensitisation. However, a significant milestone will be overcoming the obstacles precluding women from reliably self-collecting their samples for testing without third-party help and possibly in the comfort of their homes.

Self-sampling and subsequent HPV testing could be a great strategy to improve uptake and participation in screening to reduce the burden on LMICs, especially in climes where the culture or geographic location restricts women’s access to healthcare services [16]. The self-sampling method is reportedly reliable and accurate like the medic-collected samples [10, 17]. Also, HPV testing on self-collected and medic-collected samples showed comparable performance, particularly for nucleic acid amplification assays [13, 21, 22]. Self-sampling for HPV DNA testing provides an alternative to medic-sampling and should improve the uptake of HPV DNA testing in Nigeria [7, 18–23]. Studies on the acceptance of self-sampling by women showed positive results [24–27]. HPV DNA testing coupled with self-sampling methods will increase the uptake of HPV testing and provide results to ensure a good prognosis among women from low socioeconomic and minority populations [13]. Desai and colleagues reported that 82% of women (30-49 yrs) who participated in a community HPV testing in Southwestern Nigeria preferred self-sampling, leading to an increase in HPV screening [27]. Also, the self-sample collection increased the uptake of HPV screening among women (30-65 yrs) in a semi-urban community in Northcentral Nigeria, where cultural norms restrict women’s free access to healthcare services [18]. However, the two studies did not compare the test outcome of the self-collected sample pairwise with that of the medic-collection for the same individuals. Pairwise comparison is crucial to establish that the self-sampling method gives accurate and sensitive test results compared to samples collected by healthcare professionals.

This study, therefore, assessed the efficiency of a self-collected vaginal swab sample vs a medic-collected vaginal swab sample for HPV screening among sexually active women at the outpatient antiretroviral therapy centre of the Nigerian Institute of Medical Research, Lagos, Nigeria.

## Methods

### Study design

This study was a cross-sectional comparative study. Following ethical clearances from both the Institutional Review Board of the Nigerian Institute of Medical Research (NIMR; IRB/20/008) and the Chinese Centre for Disease Control and Prevention (China CDC; No. 202111), we serially recruited sexually active women at the NIMR’s antiretroviral therapy (ART) clinic and outpatient clinic in Lagos, Nigeria from March 2020 to August 2020. The ART clinic catered for HIV-positive women, while the outpatient clinic catered for staff, families, and patients attending our viral hepatitis and hypertension clinics. The research team, comprising of the clinicians, nurses, counsellors, and basic scientists approached the women at different contact points within the clinics. Women living with HIV (WLHIV) were recruited at the ART clinic, while HIV-negative women were recruited from the outpatient clinic in NIMR. WLHIV were targeted for this study due to the high prevalence of HPV among them, ensuring we will obtain positive cases to enable an balanced evaluation of the self-sampling method.

Assuming the prevalence of HPV positive in women in Nigeria to be 18%, with the significance level set at 5% and admissible error at 10%, sensitivity and specificity of self-sampling corresponding to medic-sampling were both 0.9. The formula below calculated the smallest sample size to be 192.

$$N=\frac{\left[{Z}^2\ast S\ast \left(1-S\right)\right]}{P\ast {E}^2}$$

N = Minimum sample size

$$N=\frac{1.96^2\ast 0.9\ast \left(1-0.9\right)}{0.1^2\ast 0.18}$$

Z = normal deviate for two-tailed alternative hypothesis at a 95% level of significance = 1.96.

S = Expected sensitivity and specificity of self-sampling method = 0.9.

P = Prevalence of HPV among women in Nigeria = 18%.

E - Margin of error (10%).

All participants gave informed consent. Sexual activity and mensurating status were determined by self-reporting. After explaining the study to the women, those who had abstained from sex in the last 24 hours could go for sampling immediately. Others, who were eligible and willing but either had sex within the last 24 hours or were mensurating, rescheduled their sampling till their next clinic visit. We excluded women who had undergone total hysterectomy, pregnant women, sexually inactive women and menstruating women – actively shedding uterine linings. According to the manufacturer’s protocol, the haemoglobin from menstruation can affect Polymerase Chain Reaction (PCR). Thus, women menstruating within the last 3 days before sample collection were excluded.

### Data collection

#### Procedure for sample collection

Clinicians, nurses, and health care workers trained to collect cervical swab samples were involved in collecting the medic samples. We used the sampling kit for both samplings, and participants abstained from sexual activity for a minimum of 24 hours before sample collection. Medical personnel collected samples using a speculum. Each participant received another labelled sampling kit to self-collect another sample on the same day, guided by a poster with full pictorial descriptions of the self-collection procedure (supplementary Fig. 1). Swabs were returned to the collection tube containing the preservation fluid and capped before submitting to the study team, who brings the samples collected to the laboratory daily. Both samples were obtained from patients early in the day.

#### Laboratory testing

The testing laboratory in NIMR received the swab samples daily, and they were stored at + 2 °C for immediate testing or at − 20 °C for testing later. The laboratory tested for HPV using the 15 High-risk Human Papillomavirus DNA Genotyping Diagnostic Kit (Polymerase Chain Reaction-Fluorescence Probing) on an Iponatic 96 equipment. Briefly, 20 μl sample, 10 μl lysis buffer, 30 μl PCR-mix, and 2 μl enzyme were added into predefined tubes as directed by the manufacturer’s protocol. Each set of tubes was analysed on the Iponatic 96 system, performing sample preparation and real-time PCR using four channels (FAM/CY5/ROX/HEX) within 30 minutes.

The assay employs real-time PCR to detect four targets, HPV 16, HPV 18, “Other High-Risk” (OHR), and the human β-globin as an internal control. The Iponatic 96 detects HPV18 on the FAM channel, HPV16 on the CY5 channel, OHR HPV on the ROX channel, and the internal control on the VIC/HEX channel. If the internal control is not detected, the assay returns an “invalid” result. Following a successful run on the Iponatic 96 system, possible results include HPV negative or positive for HPV 16, HPV 18, OHR, or any mixture of the three. The assay has a cyclic threshold (Ct) of ≤40 for the internal gene and a Ct of ≤39 for HPV targets. The result is invalid if a sample does not have the required Ct for the internal gene. The internal control is a human marker, thus controlling for proper sample collection, extraction, and amplification.

### Statistical analysis

Epi Info 7 was used to manage and analyse study data. The sensitivity, specificity, and accuracy of the self-collected samples’ were determined with MedCalc Online Diagnostic Calculator, using the medic-collected sample values as reference. The figures were generated using the R-program (version 4.1.1).

## Results

Two hundred and thirteen participants were recruited for this study, and their ages ranged from 16 to 63 years, with a median age of 40. With the medic-collected 213 samples, four (1.9%) samples returned invalid due to non-detection of the internal gene. Forty-four (21.1%; 44/209) samples were positive for HPV while 165 (79.0%; 165/209) were HPV negative. We detected mono infections with HPV16, HPV18 and OHR in two, five and 35 persons, respectively (Table 1). The median age for HPV positive and negative was 39 and 40 years old, respectively, with an ANOVA *p*-value of 0.3932. The stratification of HPV infection (+/−) by the age group was not statistically significant (*P* > 0.05).

**Table 1:** HPV test result from the medic-collected sample

HPV Test Results	HPV Positive				HPV Negative	Invalid	Total
	HPV 16	HPV 18	HPV 18 & OHR	HPV OHR			
n(%)	2(0.94)	5(2.35)	2(0.94)	35(16.43)	165(77.46)	4(1.88)	213(100)

**Fig. 1:**
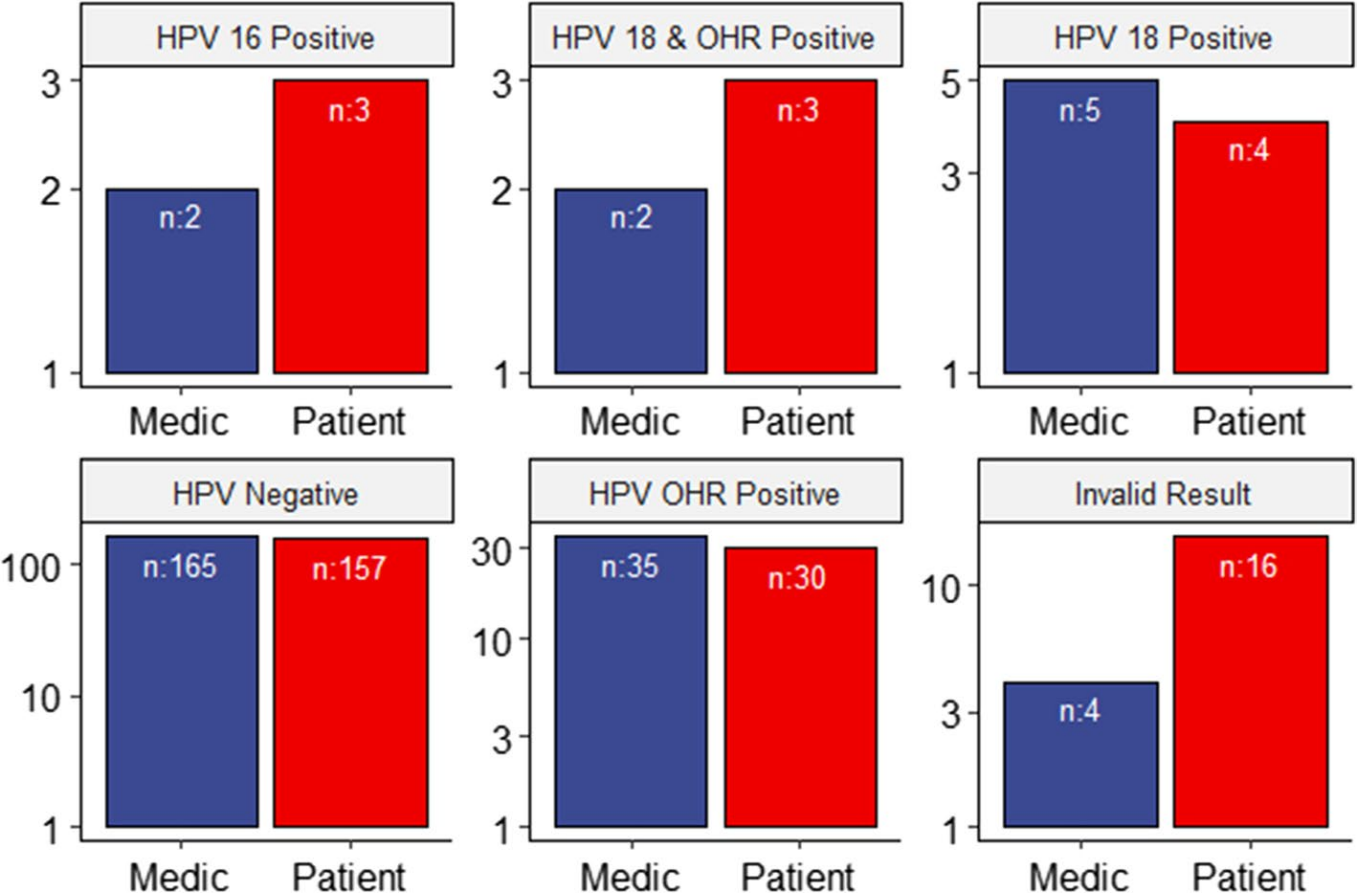
Comparison of HPV DNA Test results by the sampling methods

One hundred eighty seven (87.79%, *n* = 213) persons had concordant test results between the medic-collected and self-collected samples, while 26 (12.20%, n = 213) had discordant results. Two samples were invalid in both the medic- and self-collected samples, while 35 and 150 were concordant HPV positive and negative, respectively. Self-collected samples had more HPV16 positive (1 sample), HPV18 together with OHR positive (1 sample) and invalid results (12 samples) compared to the medic-collected ones. The medic-collected samples had more HPV 18 positive (1 sample), HPV negative (8 samples) and HPV OHR positive (5 samples) results compared to the patient-collected ones. Twenty seven out of the 35 OHR positive were concordant on both patient and medic-collected samples. In contrast, eight samples were positive on the medic-collected sampling but either negative, invalid or HPV16 positive on the self-collected samples. Thus, on both the self and medic-collected samples, there were 27 paired concordant HPV-OHR positive samples and eight discordant samples (Fig. 1).

Of the 26 (12.20%, n = 213) samples with discordant results, 14 had valid results with the medic-collected samples, while the self-collected samples were invalid (Table 2). Furthermore, some samples reported as positive from the medic-collected sample were positive for different HPV strains or negative when tested with the self-collected sample. Surprisingly, three HPV-positive samples from self-collection had their medic-collected pair negative for all strains of HPV (Table 2).

**Table 2:** Discordant HPV Test Results between Self- and Medic-collected samples

		Self-collected sample results						Total
		HPV 16 +	HPV 18 +	HPV 18 & OHR +	HPV OHR +	HPV -	Invalid	
Medic-collected sample results	HPV 16 +	0	0	0	0	0	0	0
	HPV 18 +	0	0	1	0	0	0	1
	HPV 18 & OHR +	0	0	0	0	0	0	0
	HPV OHR +	1	0	0	0	5	2	8
	HPV -	0	0	0	3	0	12	15
	Invalid	0	0	0	0	2	0	2
	Total	1	0	1	3	7	14	26

The sensitivity of the self-collected sample compared to the medic-collected samples was 89.80% (95% CI: 77.77 ~ 96.60%), while the specificity was 98.21% (95% CI: 94.87 ~ 99.63%). The accuracy of the self-collected sample using the medic-collected sample as a reference was 96.31% (95% CI: 92.87 ~ 98.40%).

## Discussion

This comparative study examined the concordance between paired self-collected and medic-collected vaginal swab samples among 213 women. The involvement of WLHIV as participants in this study was necessary to ensure we obtain HPV-positive cases for the evaluation of the self-sampling against medic-sampling. Conversely, HIV-negative women were also recruited to get HPV-negative samples for the paired analysis. Since none of the study participants had ever tested for HPV DNA, we implemented these considerations to ensure sufficient HPV-positive and HPV-negative samples for the evaluation.

Of the 213 paired samples, 187 (87.8%) samples were concordant, while 26 showed a disparity in results. This finding is comparable to the reports from other studies, showing self-sampling performs well, especially for HPV DNA testing, and can improve screening coverage [15–21, 28]. Meanwhile, the self-sampling method has a higher specificity than sensitivity when using the medic-sampling method as standard as in this study, demonstrating a comparatively low error in judgement concerning HPV infection.

Concordant invalid tests for two samples indicate the utility of the internal control in assuring the sample collection, extraction, and testing procedures. Failing to detect the internal control will prompt the repeat testing of samples not well collected and the possible identification of vaginal products interfering with PCR. The utility of the internal control will be critical if self-sampling is scaled up as the method of choice, improving test reliability and assuring caregivers.

The disparities observed include 14 self-collected samples returning invalid results while their medic-collected pair had a valid result. Seven samples tested negative on the self-collected sample, while five of their medic-collected pair were HPV positive and two invalids. Three tested positive on self-collected but negative on medic collected, and two tested positive for different HPV strains using the paired samples. Five of the samples testing invalid from the self-collected group had returned the swab sample empty as they inadvertently discarded the liquid preservative in the tube, which could be responsible for the invalid result. This occurrence can be mitigated with improved guidance documents and appropriate graphics to support the self-collection procedure. The point-of-care testing (POCT) system we used in this study targets four biomarkers, gives results within 30 minutes and does not require extraction. So, it is suitable for low throughput settings such as a doctor’s office to facilitate same-day testing and treatment.

We also reported a higher prevalence of OHR HPV-infected persons than infection with HPV 16 or 18. This finding is comparable to the report of Ajenifuja et al., finding HPV 58 as the most common strain in another city in South Western Nigeria [7]. With an accuracy of 96%, self-sampling can improve the uptake and coverage of HPV DNA testing among women across different cultures and geographic locations in Nigeria. Given the long-term latency of HPV infection, early detection and prompt availability of results using the POCT assay will enable the implementation of relevant interventions.

Although the cost of HPV DNA testing is one of the barriers of cervical cancer screening, especially in the LMICs, the cost of the assay used in this investigation is similar to the general HPV RT-PCR assays available in the Nigerian market. Therefore, beyond the self-sampling method, researchers should also innovate low-cost, flexible, sensitive, and precise assays that can encourage women of low financial income to partake in HPV DNA testing.

One of the limitations of this investigation is that a small number of participants were recruited, basically evaluating the feasibility of implementing the self-collected sampling method. There may be a need to confirm the outcome using a larger cohort and across the six geographical zones in Nigeria. Furthermore, there was no cytology or histology testing of positive samples during this study, which could have been confirmatory of possible morphological changes. Further research could include deploying self-testing in hard-to-reach communities and evaluating its impact on the uptake of HPV screening, early detection and treatment leading to a low incidence of cervical cancers in women living in Nigeria.

## Conclusion

With a sensitivity, specificity, and accuracy of 89, 98 and 96%, respectively, self-sampling is effective and if implemented may improve the uptake and coverage of HPV DNA testing among women, especially in hard-to-reach communities. Though more patient-collected samples resulted in invalid tests, most likely due to poor collection, they could be quickly identified for repeat testing, and future implementation can avoid this error with improved guidance and awareness.

## Supplementary Information

**Additional file 1.**

## Data Availability

All data generated or analysed during this study are included in this published article.

## Abbreviations

ART: Antiretroviral therapy
China CDC: Chinese Center for Disease Control and Prevention
DNA: Deoxyribonucleic acid
HICs: High-income countries
HPV: Human Papillomavirus
LMICs: Low and middle-income countries
NIMR: Nigerian Institute of Medical Research
OHR: Other High Risk
POCT: Point-of-care testing
VIA: Visual inspection under acetic acid
