# Supplementary material for: Self-collected versus medic-collected sampling for human papillomavirus testing among women in Lagos, Nigeria: a comparative study
Source: BMC Public Health. 2022 Oct 15;22:1922. doi: 10.1186/s12889-022-14222-5 (PMC9569041; doi:10.1186/s12889-022-14222-5)
Supplement: Supplementary file 1 — Additional file 1. [file 12889_2022_14222_MOESM1_ESM.zip › POPGEC Group Consortium.pdf]

Population Genomics and Cancer (POPGEC) Group, Centre for Human Virology and Genomics, Nigerian Institute of Medical Research, Lagos, Nigeria.

The POPGEC Group is led by Dr C.K. Onwuamah, involving project staff, interns and student trainees. The last two cadre is always evolving as interns/trainees finish their programme with the group. Consequently, the team list is always changing. Many were involved at different stages but not sufficient to be authors. Those whose contributions suffice to be authors are listed along with the Group project staff. We have made efforts to acknowledge all who engaged with the team activities within the various stages of study implementation and data analysis.

Group Members listed as authors

UWANDU Mabel	Nigerian Institute of Medical Research
ABIMBOLA Bowofoluwa Sharon	Nigerian Institute of Medical Research
VINCENT Grace Deborah	Nigerian Institute of Medical Research
OKOLI Leona Chika	Nigerian Institute of Medical Research
ADESINA Mary	Nigerian Institute of Medical Research
AHMED Rahaman Ademolu	Nigerian Institute of Medical Research
SOKEI Judith	Nigerian Institute of Medical Research
MOMOH Abidemi Esther	Nigerian Institute of Medical Research
SOWUNMI Omowunmi	Nigerian Institute of Medical Research
LABO-POPOOLA Olaoniye Habeebat	Nigerian Institute of Medical Research

Group Members not listed as authors

SUNDAY Mfon Victoria	Nigerian Institute of Medical Research
FAYEMI Janet	Nigerian Institute of Medical Research
UDOH Hannah Mfon	Nigerian Institute of Medical Research
OMIDIJI Mayokun	Nigerian Institute of Medical Research
OGUNDEPO Oluwatobi	Nigerian Institute of Medical Research
OGBOLU Victor	Nigerian Institute of Medical Research

Contact:

MOMOH Esther popgec2020@gmail.com;

ONWUAMAH Chika Kingsley ck.onwuamah@nimr.gov.ng; chikaonwuamah@yahoo.com
