# Supplementary material for: Self-collected versus medic-collected sampling for human papillomavirus testing among women in Lagos, Nigeria: a comparative study
Source: BMC Public Health. 2022 Oct 15;22:1922. doi: 10.1186/s12889-022-14222-5 (PMC9569041; doi:10.1186/s12889-022-14222-5)
Supplement: Supplementary file 1 — Additional file 1. [file 12889_2022_14222_MOESM1_ESM.zip › Poster.pdf]

CERVICAL CANCER
IS THE
2ND MOST COMMON CANCER
IN WOMEN LIVING IN LOW- & MIDDLE-INCOME COUNTRIES

Good News!

It is 100% preventable if detected early.

Self-testing is one of the ways to prevent cervical cancer

Common symptoms:

- **Abnormal vaginal bleeding**
- **Heavier and longer menstrual cycle**
- **Unusual vagina discharge**
- **Discomfort/pain during urination**
- **Pain during sex**
- **Waist pain**
- **Leg pain**
- **Loss of bladder control**

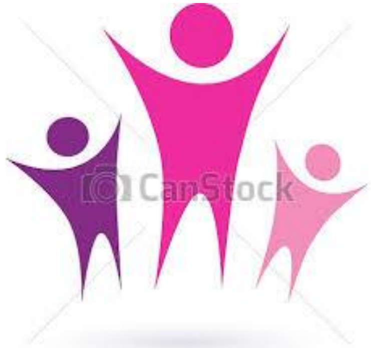

YOU CAN DO FREE SELF TESTING HERE!

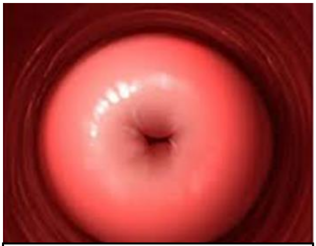

Healthy cervix

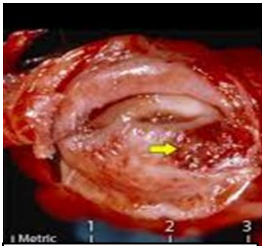

Cancer in cervix

Sample Collection Kit User Guide

1. Do not use intravaginal medication or rinse the vagina for 3 days
2. No sexual activity within 24 hours
3. Examination should be performed during non-menstrual periods
4. Recommended sample storage conditions：
   a. Samples are stored at room temperature (18-25 ° C) for 1 weeks
   b. Samples are stored for 1 month at 2-8 ° C
   c. Samples are stored for 4 months at -20 ° C
   d. Note：Avoid repeated freeze-thaw cycles

Participant Preparation

1. Introduce the study to the woman, the woman reviews the informed consent form and signs. Administer the study questionnaire completely.
2. Paste study patient code number on the study questionnaire and informed consent. Fill in the information on the sample collection kit tube or card.
3. Take out DNA sample preservation tube or card and paste study patient code number on the tube or card.
4. Discard the desiccant in the aluminum foil bag.

Clinician Sampling the Study participant

1. The woman lies on her back on the examination bed with her legs up and separated.
2. Place the head of the sampling brush at the vaginal orifice, and slowly push it into the vagina until it reaches 1/2 - 1/3 of the brush holder.
3. Twist the brush holder in the same direction for 3-5 turns, and then slowly take out the sampling brush.
4. **If using the card**, apply the sampling brush repeatedly in the middle area of the DNA sample preservation card until it changes color.
5. The DNA sample preservation card is left to dry for 2-5 minutes and sealed in an aluminum foil bag.
6. **If using the tube**, put the sampling brush into the preservation fluid and break off the handle at the breakpoint. Screw the tube cover on.
7. Package the questionnaire and sample collection kit ready for pick-up.

1 **Raise your leg**

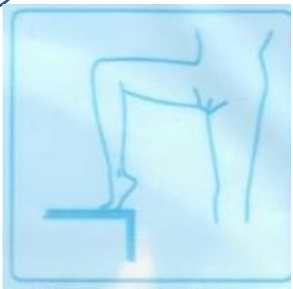

2 **Gently Insert the brush into your vagina**

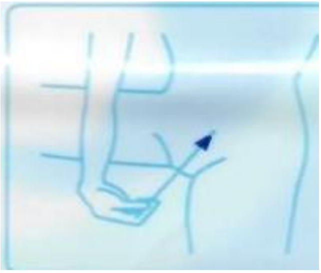

3 **Rotate the brush gently 1-3 times**

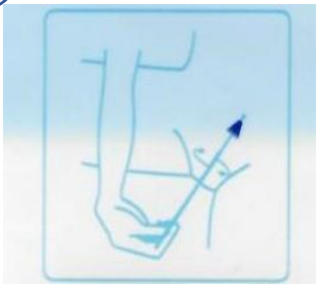

4. Remove the brush and place it in the tube

5. Return the tube to the doctor/nurse
