# Supplementary material for: Self-collected versus medic-collected sampling for human papillomavirus testing among women in Lagos, Nigeria: a comparative study
Source: BMC Public Health. 2022 Oct 15;22:1922. doi: 10.1186/s12889-022-14222-5 (PMC9569041; doi:10.1186/s12889-022-14222-5)
Supplement: Supplementary file 1 — Additional file 1. [file 12889_2022_14222_MOESM1_ESM.zip › Sansure-Flyer-HPV.pdf]

Sansure

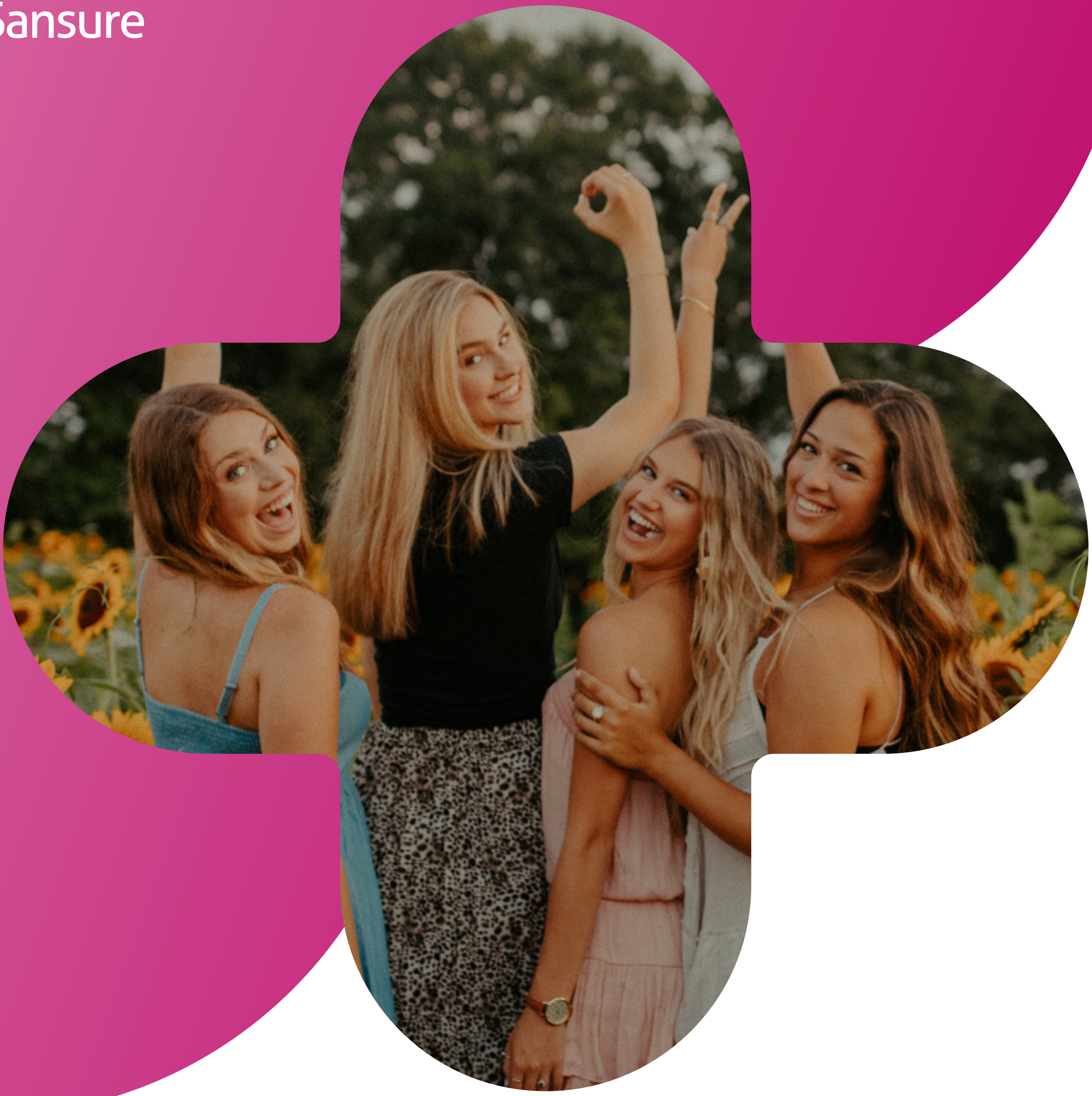

Accurate test for women's healthcare

Cervical cancer screening solutions

HPV DNA detection product series (PCR-Fluorescence Probing)

NOTICE
THIS BROCHURE IS FOR REFERENCE PURPOSES ONLY AND IS SUBJECT TO CHANGE WITHOUT NOTICE.

DISEASE BACKGROUND

- Cervical cancer is caused by sexually acquired infection with certain types of HPV.
- Two HPV types (16 and 18) cause 70% of cervical cancers and pre-cancerous cervical lesions.
- Cervical cancer is the fourth most common cancer among women globally, with an estimated 570,000 new cases in 2018.
- **Screening** and treatment of pre-cancer lesions in women is a cost-effective way to prevent cervical cancer.
- HPV DNA testing for **high-risk HPV** types is one of types for screening tests that are currently recommended by WHO.

Source : World Health Organization[1]

Estimated age-standardized incidence rates (World) in 2020,cervix uteri, all ages[2]

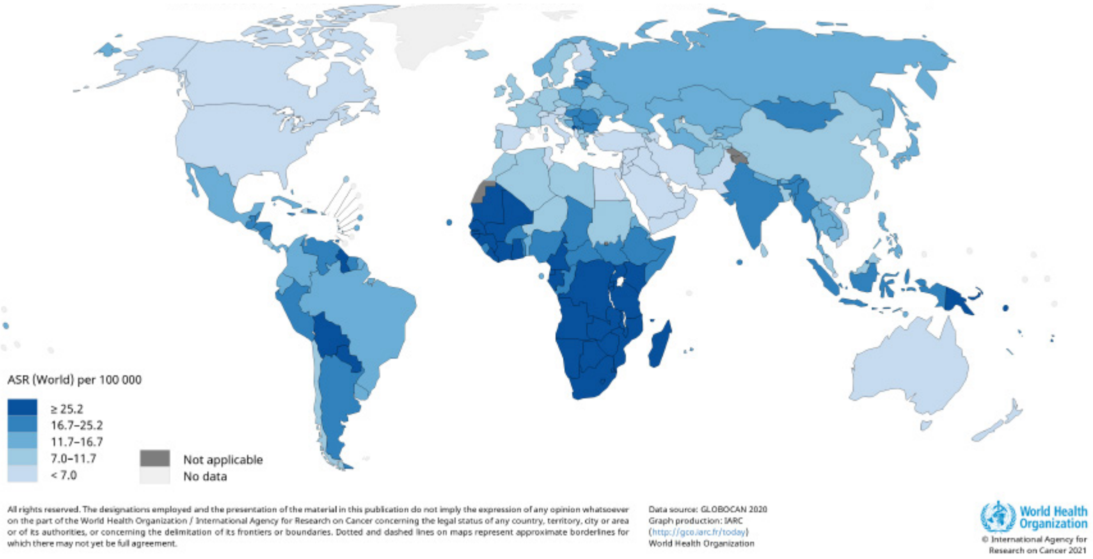

References
(1) https://www.who.int/news-room/fact-sheets/detail/human-papillomavirus-(hpv)-and-cervical-cancer
Date accessed: 2020-11-11
(2) https://gco.iarc.fr/today/home
Date accessed: GLOBOCAN 2020

Sansure
SANSURE BIOTECH INC.
ADDR. : No. 680, Lusong Road, Yuelu District, Changsha, Hunan Province, 410205, P.R.China
TEL. : +86-731-88883176-6116
FAX : +86-731-89736985
MAIL: info@sansure.com.cn / support@sansure.com.cn
WEB: eng.sansure.com.cn

SCAN ME

NOTICE THIS BROCHURE IS FOR REFERENCE PURPOSES ONLY AND IS SUBJECT TO CHANGE WITHOUT NOTICE.

HPV DNA EXTRACTION AND PURIFICATION TECHNOLOGY

One-tube fast release technology

one-tube fast release technology is the world's leading PCR detection technology in the industry. Adopting Sansure patent nucleic acid release technology, can quickly lyse pathogens at room temperature, no need heating, centrifuging or replacing tubes , the sample DNA/RNA can be extracted quickly through simple operations, combining with efficient amplification system, to realize high sensitivity and wide linear range RT-PCR detection with good reproducibility , strong anti-interference ability and multiple genotypes coverage.

WORKFLOW

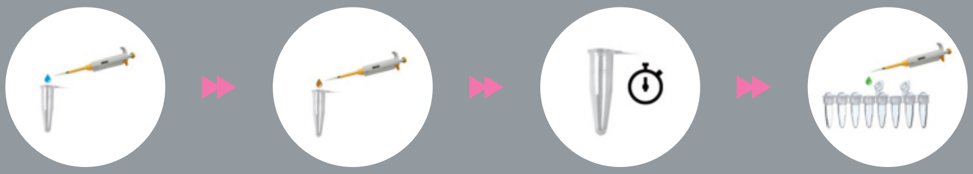

Add lysis buffer → Add sample → 10min. room temperature lysis → Add PCR master mix ready for PCRdetection

Advanced magnetic beads technology

Since 2008, Sansure new generation "advanced magnetic beads technology" using exclusive modified super-paramagnetic nano-beads to absorb sample DNA/RNA , only needs a simple step of washing to obtain high purity nucleic acid, combined with the "DNA/RNA elution-free " technology and integrate efficient amplification system, to achieve DNA/RNA amplification detection with magnatic beads. It can realize high sensitivity and wide linear range PCR detection with good reproducibility , strong anti-interference ability and multiple genotypes coverage.

WORKFLOW

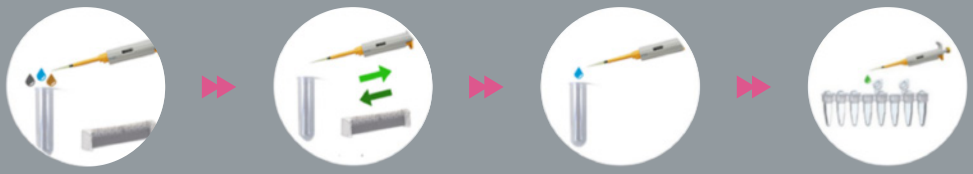

Add lysis buffer, sample, magnetic beads at room temperature → Add washing buffer 1&2 One time washing for purification → Add PCR mix → Transfer template and PCR mix ready for real time PCR

HPV DNA DETECTION WORKFLOW

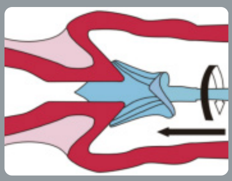

Sample collection

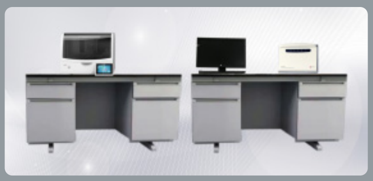

HPV DNA detection

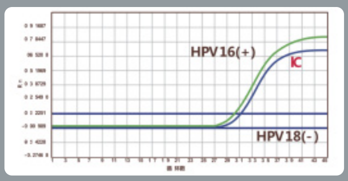

Result analysis

Sansure
SANSURE BIOTECH INC.

ADDR. : No. 680, Lusong Road, Yuelu District, Changsha, Hunan Province, 410205, P.R.China
TEL. : +86-731-88883176-6116
FAX : +86-731-89736985
MAIL: info@sansure.com.cn / support@sansure.com.cn
WEB: eng.sansure.com.cn

SCAN ME

NOTICE THIS BROCHURE IS FOR REFERENCE PURPOSES ONLY AND IS SUBJECT TO CHANGE WITHOUT NOTICE.

ADVANTAGES

ONE STEP, SIMPLIFIED
No heating, centrifugation, hybridization, contamination avoidance, simplified operation.

OPEN PLATFORM
Based on RT-PCR technology, applicable to mainstream types of PCR instrument.

FULLY AUTOMATED, HIGH-THROUGHPUT
Matching with the Natch CS/CS2 fully automated nucleic acid extraction system.

QUALITY CONTROL
UNG enzyme + dUTP system to prevent and control aerosol contamination .
β-globin internal control, monitoring the whole process, avoiding false negative.

15 COMMON HIGH-RISK TYPES
covering more than 99% of high-risk types.

COMPREHENSIVE CLINICAL APPLICATION
Cervical cancer screening, colposcopy indication judgment, ASCUS shunt, cervical lesions follow-up, guidance of vaccine use, genital condyloma acuminatum assisted diagnosis.

PARAMETERS

Product features	Parameter
Specimen Type	Exfoliated cells from females' cervix
Technical Platform	One-tube fast release technology<br>Advanced magnetic beads technology
Detection Types	Type 16, 18, 31, 33, 35, 39, 45, 51, 52, 53, 56, 58, 59, 66, 68
Internal Control	β-globin gene
Quality Control	UNG enzyme + dUTP anti-contamination system
Compatible Instrument	SLAN-96P, Roche 480 and ABI 7500
Amplification Time	70 min
Sensitivity	400 copies/mL for S3027E, S3019E, S3017E<br>1000 copies/mL for S3031E, S3057E

Sansure
SANSURE BIOTECH INC.

ADDR. : No. 680, Lusong Road, Yuelu District, Changsha, Hunan Province, 410205, P.R.China
TEL. : +86-731-88883176-6116
FAX : +86-731-89736985
MAIL: info@sansure.com.cn / support@sansure.com.cn
WEB: eng.sansure.com.cn

SCAN ME

NOTICE THIS BROCHURE IS FOR REFERENCE PURPOSES ONLY AND IS SUBJECT TO CHANGE WITHOUT NOTICE.

SANSURE HPV DNA DETECTION SOLUTIONS

1.MANUAL SOLUTION

Simple operation, Heating-free extraction, no tube changing needed.
Fast and accurate detection, can be processed 96 samples at one time.

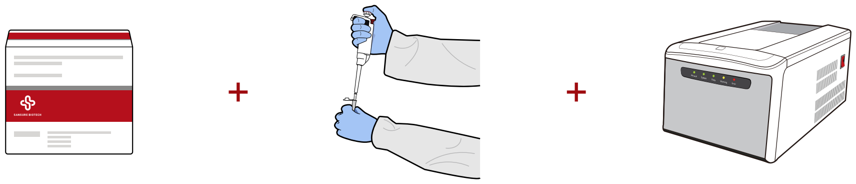

2.FULLY AUTOMATED SOLUTION

Samples can be processed in batches to avoid manual errors and exposure risks. It features high-throughput and high-efficiency. The fully automatic nucleic acid extraction instrument can process 96 samples in 90 minutes.

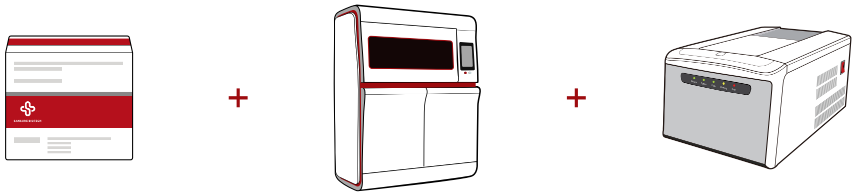

3. TABLE-AUTOMATED SOLUTION

High-throughput and high-efficiency
The automatic nucleic acid extraction instrument can process 1-96 samples in 16 minutes.

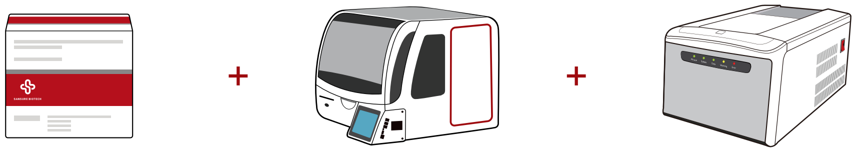

4. iPonatic POCT SOLUTION

Time-saver: starting from the sample entry, the test results can be read in 8-30 minutes
Easy operation: no heating, no manual operation, automatic sample entry.

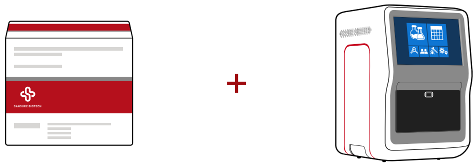

Sansure
SANSURE BIOTECH INC.

ADDR. : No. 680, Lusong Road, Yuelu District, Changsha, Hunan Province, 410205, P.R.China
TEL. : +86-731-88883176-6116
FAX : +86-731-89736985
MAIL: info@sansure.com.cn / support@sansure.com.cn
WEB: eng.sansure.com.cn

SCAN ME

NOTICE THIS BROCHURE IS FOR REFERENCE PURPOSES ONLY AND IS SUBJECT TO CHANGE WITHOUT NOTICE.

EXTRACTION AND AMPLIFICATION KITS

Item No.	Product Name	Spec.
S3057E	Human Papillomavirus DNA Diagnostic Kit (PCR-Fluorescence Probing)	48T/kit
S3031E	15 High-risk Human Papillomavirus DNA Diagnostic Kit (PCR-Fluorescence Probing)	48T/kit
S3027E	High-risk Human Papillomavirus DNA (Genotype) Diagnostic Kit (PCR-Fluorescence Probing)	24T, 48T/kit
S3019E	Human papillomavirus (Type 16 and 18) DNA Fluorescence Diagnostic Kit (PCR- Fluorescence Probing)	48T/kit
S3017E	Human papillomavirus (Type 6 and 11) DNA Fluorescence Diagnostic Kit (PCR-Fluorescence Probing)	48T/kit
S1013E	Sample Release Reagent	48T/kit
S1006E	Multi-type Sample DNA/RNA Extraction-Purification Kit (Magnetic beads method)	48T/kit
S10016E	Nucleic Acid Extraction-Purification Kit (Magnetic beads method)	48T/kit

EXTRACTION AND AMPLIFICATION INSTRUMENTS

Model No.	Product Name
Natch48	Nucleic Acid Extraction System
S12C	Fully Automated Nucleic Acid Extraction System (Natch CS)
S-S13A	Fully Automated Nucleic Acid Extraction System (Natch CS2)
SLAN-96P	Real-Time PCR System (SLAN-96P)
MA-6000	Real-Time Quantitative Thermal Cycler (MA6000)
S-Q31A	Portable Molecule Workstation (iPonatic 1 module)
S-Q31B	Portable Molecule Workstation (iPonatic 4 modules)

All products above are NMPA approved and CE marked

Sansure
SANSURE BIOTECH INC.

ADDR. : No. 680, Lusong Road, Yuelu District, Changsha, Hunan Province, 410205, P.R.China
TEL. : +86-731-88883176-6116
FAX : +86-731-89736985
MAIL: info@sansure.com.cn / support@sansure.com.cn
WEB: eng.sansure.com.cn

SCAN ME

20210622
