# Supplementary figures and images for: Self-collected versus medic-collected sampling for human papillomavirus testing among women in Lagos, Nigeria: a comparative study
Source: BMC Public Health. 2022 Oct 15;22:1922. doi: 10.1186/s12889-022-14222-5 (PMC9569041; doi:10.1186/s12889-022-14222-5)

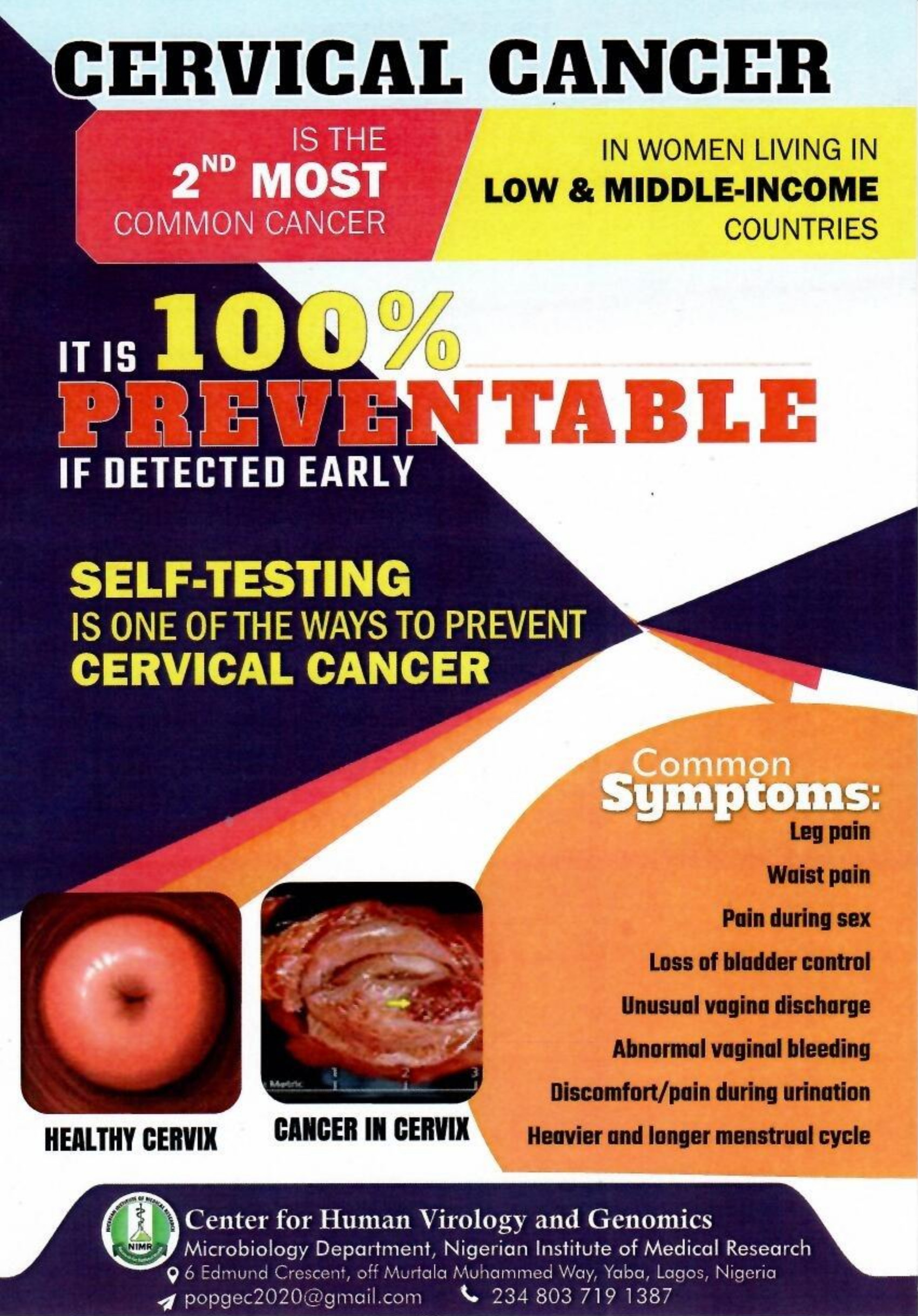

Supplement: Supplementary file 1 — Additional file 1. [file 12889_2022_14222_MOESM1_ESM.zip › HPV Study Handbill pg1.jpg]

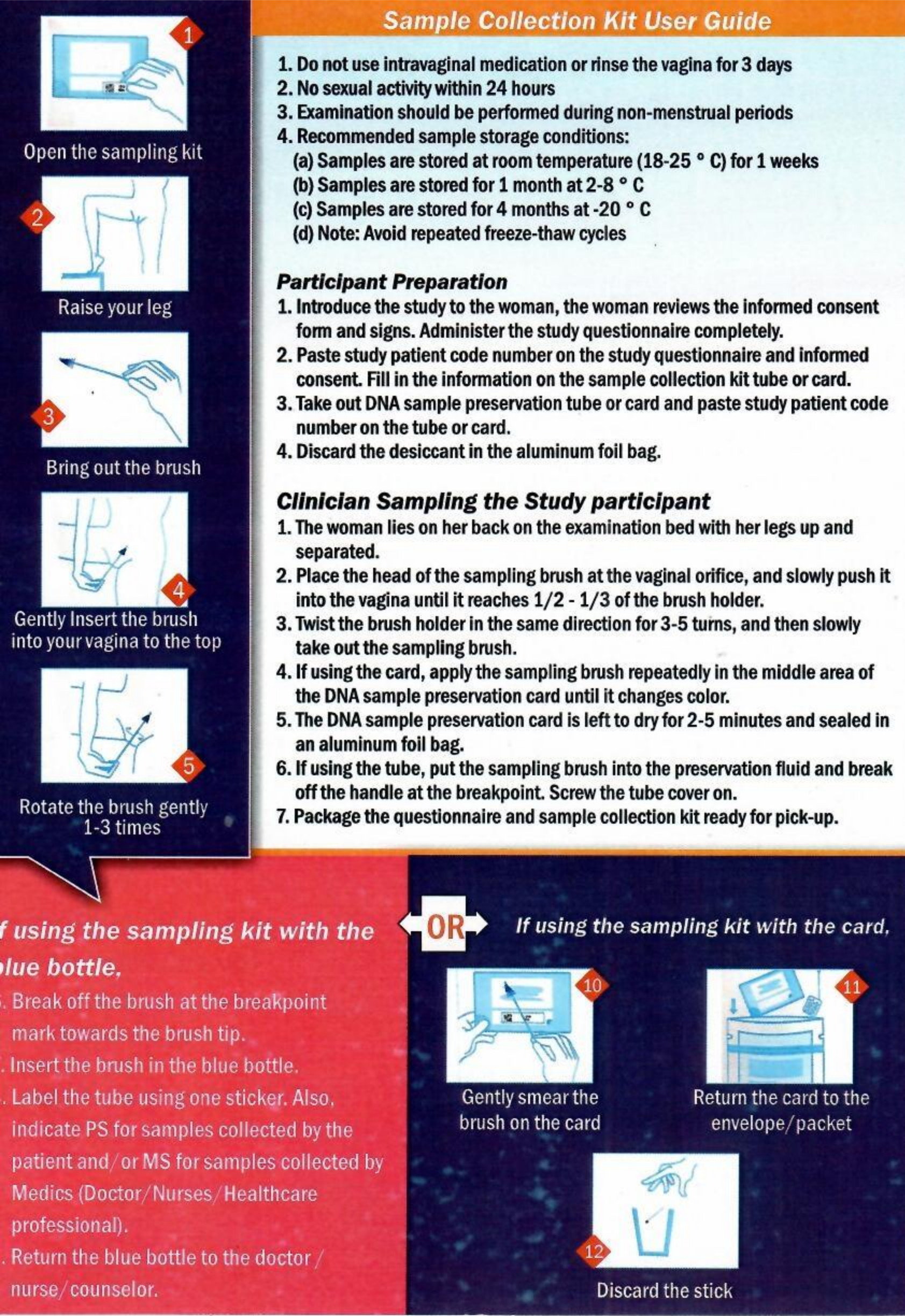

Supplement: Supplementary file 1 — Additional file 1. [file 12889_2022_14222_MOESM1_ESM.zip › HPV Study Handbill pg2.jpg]
